# Manipulating Eryptosis of Human Red Blood Cells: A Novel Antimalarial Strategy?

**DOI:** 10.3389/fcimb.2018.00419

**Published:** 2018-11-30

**Authors:** Coralie Boulet, Christian D. Doerig, Teresa G. Carvalho

**Affiliations:** ^1^Molecular Parasitology Laboratory, Department of Physiology, Anatomy and Microbiology, La Trobe University, Bundoora, VIC, Australia; ^2^Infection and Immunity Program, Biomedicine Discovery Institute, Department of Microbiology, Monash University, Clayton, VIC, Australia

**Keywords:** malaria, eryptosis, *Plasmodium*, apoptosis, programmed cell death, host-pathogen interaction, host-directed therapy

## Abstract

Malaria is a major global health burden, affecting over 200 million people worldwide. Resistance against all currently available antimalarial drugs is a growing threat, and represents a major and long-standing obstacle to malaria eradication. Like many intracellular pathogens, *Plasmodium* parasites manipulate host cell signaling pathways, in particular programmed cell death pathways. Interference with apoptotic pathways by malaria parasites is documented in the mosquito and human liver stages of infection, but little is known about this phenomenon in the erythrocytic stages. Although mature erythrocytes have lost all organelles, they display a form of programmed cell death termed eryptosis. Numerous features of eryptosis resemble those of nucleated cell apoptosis, including surface exposure of phosphatidylserine, cell shrinkage and membrane ruffling. Upon invasion, *Plasmodium* parasites induce significant stress to the host erythrocyte, while delaying the onset of eryptosis. Many eryptotic inducers appear to have a beneficial effect on the course of malaria infection in murine models, but major gaps remain in our understanding of the underlying molecular mechanisms. All currently available antimalarial drugs have parasite-encoded targets, which facilitates the emergence of resistance through selection of mutations that prevent drug-target binding. Identifying host cell factors that play a key role in parasite survival will provide new perspectives for host-directed anti-malarial chemotherapy. This review focuses on the interrelationship between *Plasmodium falciparum* and the eryptosis of its host erythrocyte. We summarize the current knowledge in this area, highlight the different schools of thoughts and existing gaps in knowledge, and discuss future perspectives for host-directed therapies in the context of antimalarial drug discovery.

## Introduction

Malaria is a vector-borne parasitic disease that affects millions of people worldwide. It is estimated that half the world population is at risk of infection and in 2016, malaria was responsible for 200 million new cases and half a million deaths (WHO|World Malaria Report, 2017). An efficient malaria vaccine has yet to be developed, and resistance against all antimalarial drugs has been recorded, including to artemisinin (reviewed in Tilley et al., 2016), the front-line drug treatment recommended by the World Health Organization (WHO). The path to controlling and eliminating malaria is a difficult one, and the systematic emergence of drug resistant parasites against every anti-malarial drug introduced over the last century represents a major road block (Menard and Dondorp, 2017). The encouraging decrease in malaria morbidity and mortality observed in recent decades has now been reported to be slowing down, and be reversing in parts of the world (WHO|World Malaria Report, 2017). To reach the strategic objective set by the WHO to reduce global malaria incidence and mortality by at least 90% by 2030 (WHO|Global Technical Strategy for Malaria, 2016–2030), new antimalarial drugs with a novel mode of action are urgently needed.

**Figure 1:**
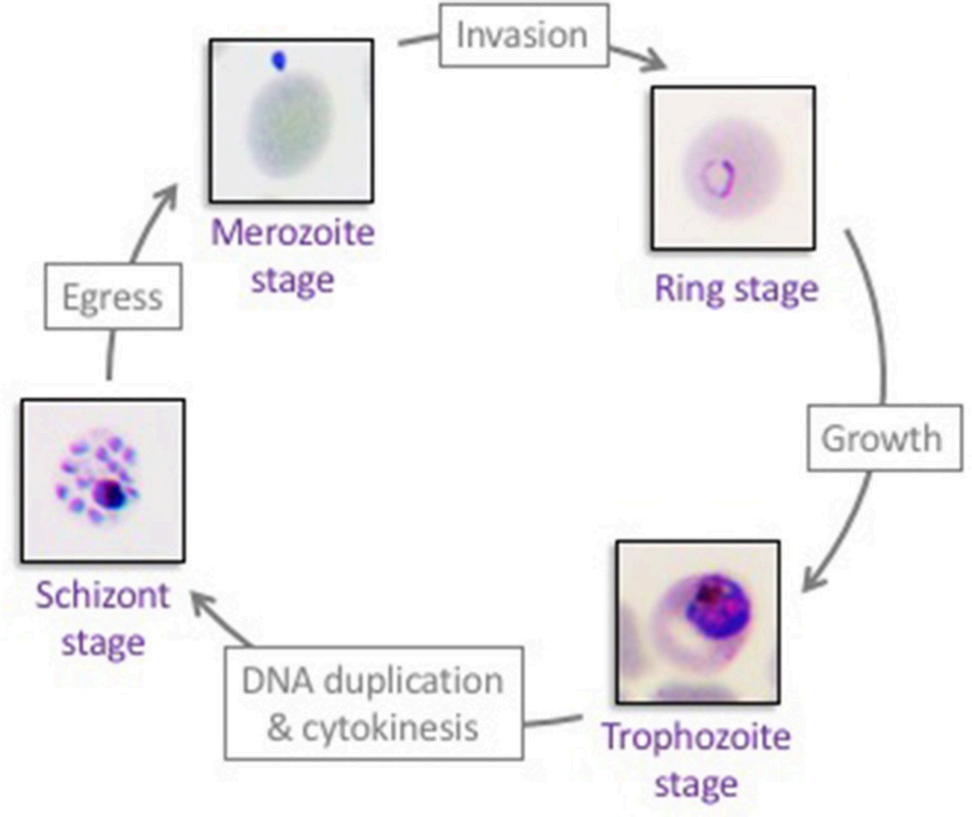
The asexual proliferation cycle of *Plasmodium falciparum* in human erythrocytes. Extracellular merozoites invade red blood cells to establish the erythrocytic asexual cycle. Each intracellular merozoite develops into an intra-erythrocytic ring stage, matures into a trophozoite stage, and subsequently forms a multi-nucleated schizont. Forty-eight hours post-merozoite infection, 8–32 new merozoites egress from each schizont-infected erythrocyte and a new erythrocytic cycle begins. Repeated cycles of erythrocyte invasion by *Plasmodium falciparum* parasites lead to all aspects of malaria pathogenesis.

Malaria is caused by the unicellular apicomplexan parasite *Plasmodium*. A human infection starts when an infected *Anopheles* mosquito injects parasites (in the form of sporozoites) during a blood meal. Sporozoites circulate in the blood stream and reach the liver, where they invade hepatocytes and establish an asymptomatic infection. *Plasmodium* hepatic stages replicate by schizogony, ultimately releasing tens of thousands progeny merozoites in the blood stream. Once in the blood stream, *Plasmodium* merozoites invade red blood cells, where they proliferate by schizogony in an asexual replication cycle, known as the erythrocytic cycle (Figure 1). The cycle begins when an extracellular merozoite invades an erythrocyte. Once intracellular, the parasite develops into a ring stage, grows into a metabolically active trophozoite, and, following DNA replication and asynchronous nuclear divisions matures into a multi-nucleated schizont. After cytokinesis, up to 32 new merozoites egress from each schizont, lysing the host red blood cell and allowing for a new cycle to begin. Alternatively, early ring stage parasites can mature into female or male gametocytes (immature sexual stage of the parasite), which, once taken up by an *Anopheles* mosquito, complete maturation and fertilization within the mosquito's gut. The resulting oocyst produces sporozoites that travel to the salivary gland of the mosquito, allowing for further transmission of the parasite. The erythrocytic stages of infection are responsible for malaria pathogenesis, whose clinical manifestations include severe anemia, organ failure and cerebral malaria (Autino et al., 2012). Among the five *Plasmodium* species that infect humans, *P. falciparum* is the most virulent. Here, we focus on host-parasite interaction mechanisms that allow the development of *P. falciparum* inside human erythrocytes (Figure 1).

## Avoiding antimalarial drug resistance by targeting the host cell?

Curative antimalarials target the asexual proliferation of parasites in erythrocytes, and all antimalarial drugs developed to date directly target parasite factors. These include artemisinin (whose mechanism of action is yet to be fully understood), chloroquine (interferes with haemozoin formation, a process that detoxifies free haem released by hemoglobin digestion), atovaquone (inhibits mitochondrial respiration), proguanil/pyrimethamine (inhibits folate biosynthesis by targeting dihydrofolate reductase, PfDHFR), and various antibiotics which inhibit protein synthesis (Antony and Parija, 2016). Parasite resistance against anti-malarial drugs is a major long-standing issue, resulting in failure of many malaria eradication attempts. For instance, in 1955, the WHO launched a Global Malaria Eradication campaign, introducing Mass Drug Administration of chloroquine, the cheapest and most widely used antimalarial drug. However, in the 1960's, chloroquine resistance was reported in various South American and South-East Asian countries, and quickly spread, reaching African nations in the early 1970's (D'Alessandro and Buttiëns, 2001). Remarkably, *all* anti-malarial drugs saw resistance emerging within a couple of years of being commercialized (McClure and Day, 2014). Alarmingly, this also includes resistance against the current front-line drug, artemisinin, commonly used in combination therapies (Menard and Dondorp, 2017). Over relatively short periods of time, *Plasmodium* parasites have acquired genetic modifications, typically point mutations or copy number variations, resulting in resistance to antimalarial drugs. Such alterations have been shown to either affect the product directly targeted by the drug, or a membrane transporter, increasing the efflux of the drug to the outside environment. Such transporters are located on the plasma membrane of the parasite (e.g., multidrug resistance-associated protein, PfMRP) or on the food vacuole of the parasite (e.g., multidrug resistant protein 1, PfMDR1) (Antony and Parija, 2016).

Considering that *Plasmodium* is an obligate intracellular pathogen, and therefore relies on host cell factors to thrive, an alternative drug target strategy based on host cell factors can be envisaged. Host factors are not under the genetic control of the parasite, therefore host-directed therapy approaches bypass the most direct path to resistance, i.e., the selection of parasite genotypes encoding a mutated drug target.

For example, the host programmed cell death pathway could be targeted. Indeed, most intracellular pathogens avoid being eliminated by the immune system by “hiding” inside a host cell, while also gaining direct access to the host cell intracellular environment. Although this is highly beneficial to the pathogen, the stress induced by infection leads the host cell to trigger a cell death response or apoptosis. As a consequence, the pathogen is required to inhibit the host cell apoptotic response to ensure host cell survival until completion of its own replication cycle. In the case of *Plasmodium*, interference with host cell apoptosis has been established in the hepatic stage of infection (van de Sand et al., 2005; Kaushansky et al., 2013). However, the existence of a similar process in the erythrocytic stage of infection remains to be explored, likely because of the unique nature of the host erythrocyte cell.

## Red blood cells—a unique cell type

Maturation of erythrocyte progenitors in the bone marrow leads to the formation of enucleated reticulocytes that are released in the blood stream. Reticulocytes mature into erythrocytes, a process involving the loss of all intracellular organelles, including the nucleus, mitochondria, and endoplasmic reticulum (Lang and Föller, 2012). In the absence of *de novo* protein biosynthesis and mitochondria (which house the TCA cycle enzymes in nucleated cells), the survival and metabolism of mature erythrocytes rely exclusively on the existing pool of proteins and on glycolysis for the production of ATP (Lang and Föller, 2012). Further, a specialized function of oxygen transporter combined with a lack of organelles, have led RBCs to develop unique mechanisms of cell survival and cell death, which we outline below.

### Erythrocytes protect themselves from oxidative stress

Red blood cells (RBCs) have a high haem iron content, essential for their role as oxygen and carbon dioxide transporters (Lang and Föller, 2012). For this reason, RBCs are constantly producing, and consequently exposed to, reactive oxygen species (ROS), including superoxide anion (O${}_{2}^{-}$), hydrogen peroxide (H_2_O_2_) and hydroxyl radicals (OH^−^) (Baynes, 2005; Cimen, 2008; Schieber and Chandel, 2014). Reactive oxygen species impose oxidative stress on the cell, due to their inherent capability to damage lipids, proteins and DNA (although the latter is irrelevant in the context of RBCs). Erythrocytes possess a number of antioxidant strategies to counteract this stress, including ROS scavengers, such as glutathione and vitamins C and E, as well as various redox enzymes such as superoxide dismutase, catalase and glutathione peroxidase (Baynes, 2005). Glutathione is a Glu-Cys-Gly tri-peptide of crucial importance to maintain the intracellular environment in a reduced state (Wu et al., 2004). The ratio between reduced glutathione (GSH) and oxidized glutathione (GSSG) is a good indicator of the cellular redox state. Glutathione is naturally most required in tissues exposed to high ROS and oxidative stress levels, such as the liver and erythrocytes (Wu et al., 2004; Lu, 2009).

### How do RBCs die? senescence vs. eryptosis

Mature RBCs have a life span of ~115 days (Franco, 2012), after which they undergo senescence, a gradual deterioration of the cell functional capacity, and clearance by the spleen. ROS accumulation has been proposed as a key life-span determinant (Hattangadi and Lodish, 2007). During this aging process, erythrocytes lose membranes through microvesiculation, become denser, intracellular enzymes display decreased activity, oxidative damages accumulate, and the membrane becomes more rigid (Lutz and Bogdanova, 2013). The Band 3 membrane protein (also called anion exchanger 1 or AE1) forms clusters (partly due to oxidation and hyperphosphorylation), that enhances deposition of complement C3 and subsequent binding of autoantibodies present in the serum, followed by clearance by macrophages (Lutz, 2004; Arese et al., 2005, p. 3; Lutz and Bogdanova, 2013). Additionally, the progressive exposure of the “eat-me” signal phosphatidylserine (PS), along with the reduced exposure of the “do-not eat-me” signal Cluster of Differentiation 47 (CD47, further discussed below), on the outside of a senescent erythrocyte further stimulate clearance by macrophages (Lutz and Bogdanova, 2013).

In addition to this type of “death by exhaustion,” RBCs can undergo a form of programmed cell death throughout their lifetime. This phenomenon was first described in 2001 (Bratosin et al., 2001) and the term eryptosis was proposed in 2005 (Lang K. et al., 2005). Eryptosis shares numerous similarities with apoptosis (Bratosin et al., 2001; Lang K. et al., 2005). Both apoptosis and eryptosis share the common purpose of destruction of damaged cells without inducing an inflammatory response (i.e., absence of cell lysis). This is particularly relevant in the case of RBC death, as the release of free hemoglobin in the blood induces renal impairment and thrombosis (Tolosano et al., 1999; Buehler et al., 2012; Schaer et al., 2013). Further, eryptotic and apoptotic cells share physiological characteristics including increased intracellular calcium concentrations, cell shrinkage, exposure of phosphatidylserine on the outer cell surface and membrane ruffling and blebbing (Bratosin et al., 2001; Lang K. et al., 2005). Like apoptosis, eryptosis can be triggered by a wide range of xenobiotics some of which have been listed in Table 1.

**Table 1:** Examples of relevant eryptosis inducers and inhibitors and their molecular targets.

	**Compound**	**Cellular target**	**References**
Eryptosis inducers	Amiodarone	Ion channel blocker (used for treatment of cardiac arrhythmias)	Nicolay et al., 2007
	Amphotericin B	Forms cation channels in membranes (used as an anti-fungal and anti-parasite)	Mahmud et al., 2009
	Anandamide	Cannabinoid receptor agonist; induces apoptosis in diverse cell types	Bentzen and Lang, 2007
	Aurothiomalate	Rheumatoid arthritis gold-containing drug	Sopjani et al., 2008
	Azathioprine	Induces apoptosis of lymphocytes (used as an immunosuppressive drug)	Geiger et al., 2008
	Chlorpromazine	Dopamine antagonist, anti-serotonergic and antihistaminic (used as an antipsychotic drug)	Akel et al., 2006
	Cyclosporine	Inhibits gene transcription in nucleated cells (used as an immunosuppressive drug)	Niemoeller et al., 2006
	Dimethylfumarate	Decreases intracellular GSH hence induces oxidative stress (used as an anti-inflammatory drug)	Ghashghaeinia et al., 2016
	Lead	Decreases erythrocytes ATP concentration, and activates erythrocyte K^+^ channels	Kempe et al., 2005
	L-NAME	Inhibits synthase of nitric oxide (NO)	Koka et al., 2008b; Nicolay et al., 2008
	Paclitaxel	Blocks mitosis and cytoskeleton organization (used as anti-cancer drugs)	Lang et al., 2006b
Eryptosis inhibitors	Amitriptyline	Inhibits sphingomyelinase (hence ceramide production)	Brand et al., 2008; Lang et al., 2015b
	Flufenamic acid	Inhibits Cl^−^ sensitive and PGE_2_-triggered Ca^2+^ entry	Kasinathan et al., 2007

*Compounds described as eryptosis inducers or inhibitors (see references therein), their cellular/molecular target and current use. These compounds have been further utilized in the context of malaria infections (see Tables 3, 4)*.

Unbalanced levels of eryptosis have been proposed to play a key role in the pathology of numerous clinical disorders, including in metabolic syndrome (Zappulla, 2008), haemolytic uremic syndromes (Lang et al., 2006a), sickle-cell disease (Lang et al., 2002), thalassemia (Lang et al., 2002), and G6PD deficiency (Lang et al., 2002). The clinical relevance of red blood cell death in health and disease, has stimulated numerous studies over the last 20 years attempting to define the cellular and molecular mechanisms of eryptosis. Although much remains to be described, the section below summarizes our current knowledge of the regulatory mechanisms of eryptosis.

## Eryptosis—what do we know about its regulatory mechanisms?

**Figure 2:**
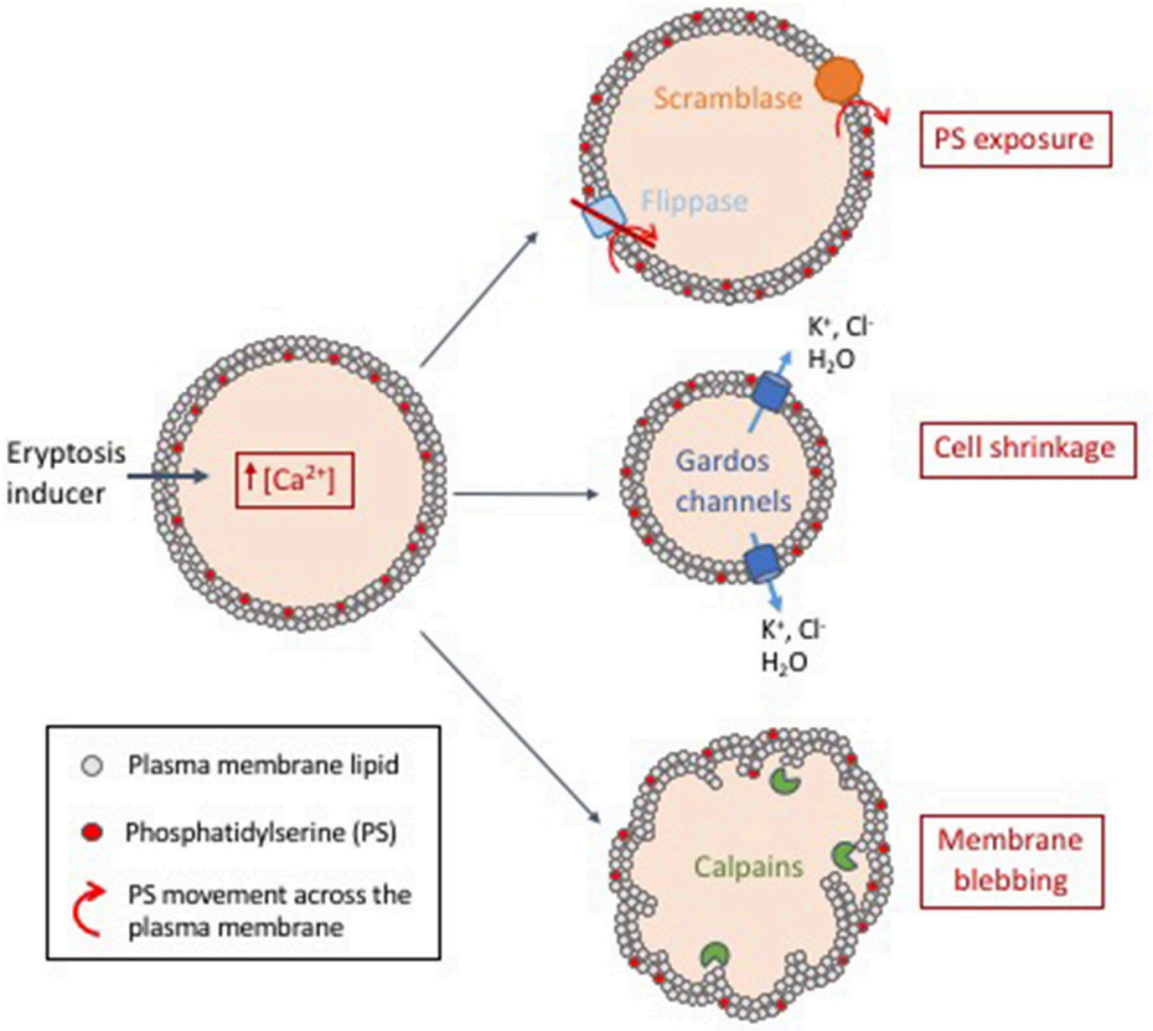
Proposed model of eryptosis mechanisms. Induction of eryptosis (energy depletion, oxidative stress or exposure to xenobiotics) leads to an increase of intracellular calcium concentration. (i) Intracellular calcium activates scramblases (membrane proteins that transport lipids non-specifically and bidirectionally) and inactivates flippases (membrane proteins that actively maintain phosphatidylserine (PS) in the membrane inner leaflet). This leads to exposure of PS at the surface of the cell, a signal that is recognized by macrophages, which then mediate clearance of eryptotic cells. (ii) Intracellular calcium activates calcium-sensitive potassium channels known as Gardos channels. This is followed by exit of potassium and chlorine, leading to loss of water by osmosis and subsequent cell shrinkage. (iii) Increased concentration of free intracellular calcium activates calpains (calcium-activated proteases). Calpains degrade cytoskeleton proteins which leads to membrane ruffling and blebbing.

Eryptosis can be triggered by various signals, including osmotic shock (Huber et al., 2001; Lang et al., 2004), energy depletion (Klarl et al., 2006), oxidative stress (Lang K. et al., 2005; Lang et al., 2014), and xenobiotics (Lang and Lang, 2015; Pretorius et al., 2016). Regardless of the trigger, induction of an eryptotic state generally involves entry of extracellular calcium ions into the cell (Lang K. S. et al., 2003), which induces changes in membrane asymmetry/exposure of PS, cell shrinkage and membrane blebbing, detailed below and summarized in Figure 2.

### Changes in cell membrane asymmetry with phosphatidylserine exposure

In normal conditions, the RBC lipid bilayer is asymmetric, with specific lipids predominantly present on the inner or the outer leaflet. This is the case of the phospholipid phosphatidylserine, which is mainly present within the cytosolic monolayer (Leventis and Grinstein, 2010). The asymmetry is maintained by active membrane proteins termed flippases, that translocate phosphatidylserine and other phospholipids from the outer to the inner membrane leaflet (Sharom, 2011; Segawa and Nagata, 2015). In eryptotic cells, however, high intracellular calcium concentrations lead to the inactivation of flippases (Suzuki et al., 2010, 2013), leading to the rupture of membrane asymmetry and exposure of PS on the outer leaflet of the RBC membrane (Bratosin et al., 2001; Segawa and Nagata, 2015). High intracellular calcium levels also lead to the activation of another family of membrane proteins termed scramblases that translocate phospholipids non-specifically and bidirectionally in an ATP-independent manner (Segawa and Nagata, 2015). In addition, caspase 3 can also cleave and thus irreversibly activate scramblases and inactivate flippases (Berg et al., 2001; Schoenwaelder et al., 2009; Suzuki et al., 2010), committing the cell to PS exposure. The increased and abnormal exposure of PS on the outer RBC membrane is recognized by macrophages (McEvoy et al., 1986), which remove eryptotic cells from the circulation (Boas et al., 1998). “Flippase” and “scramblase” are generic terms used to describe lipid-transport enzymatic activities. The exact proteins responsible for PS exposure during eryptosis remain to be identified.

### Cell shrinkage and gardos channels activation

Increased concentration of cytosolic calcium activates calcium-sensitive potassium channels, or Gardos channels (Bookchin et al., 1987; Brugnara et al., 1993), which allow the exit of K^+^ (Lang P. A. et al., 2003). The loss of intracellular K^+^ hyperpolarises the cell membrane, forcing the exit of Cl^−^ (Lang P. A. et al., 2003). Consequently, the loss of water through osmosis leads to cell shrinkage (Lang P. A. et al., 2003). Cell shrinkage activates phospholipase A, which produces platelet-activating factor (PAF) (Lang P. A. et al., 2005). PAF in turn stimulates sphingomyelinase, an enzyme that breaks down sphingomyelin (Lang P. A. et al., 2005), the most prevalent sphingolipid in the cell membrane (Barenholz and Thompson, 1980). This leads to the formation of ceramide, a well-known inducer of eryptosis (Lang et al., 2004) and apoptosis (Dbaibo et al., 1997; Birbes et al., 2002). The role of ceramide in eryptosis has been review by Lang et al. (2015b).

### Membrane blebbing and calpain activation

Increased intracellular calcium concentration leads to the activation of calpains (Berg et al., 2001), a family of cytosolic calcium-dependent thiol proteases. Calpains I (or μ-calpains) are activated by micromolar amounts of calcium, whereas activation of calpains II (or M-calpains) requires millimolar amounts of calcium (Perrin and Huttenlocher, 2002). Activated calpains target RBC cytoskeleton proteins, such as spectrin, actin, and band 3/AE1 (Murachi et al., 1981; Schwarz-Ben Meir et al., 1991), whose degradation ultimately leads to ruffling and blebbing of the RBC membrane (Larsen et al., 2008).

Depending on the eryptotic trigger, different pathways can be activated. For instance, eryptosis due to oxidative stress seems to activate caspases (Matarrese et al., 2005), although there is some controversy about the activity of caspases in RBCs (Berg et al., 2001). Interestingly, it has been observed that not all erythrocytes have the same sensitivity to oxidative stress-induced eryptosis, differences being attributed to age and oxidative stress “history” of the cell (Ghashghaeinia et al., 2012). Eryptosis triggered by energy depletion has been shown to involve a number of kinases—further discussed below—as well as an impairment in antioxidant glutathione (GSH) replenishment, which requires energy (Tang et al., 2015).

Although some of the mechanisms leading to eryptosis have been described, the molecular players and signaling pathways underpinning eryptosis remain yet to be fully characterized. So far, a few key regulators of classical apoptotic pathways, protein kinases and “death” receptors have been suggested as putative molecular regulators of eryptosis and are summarized in the next section.

## Molecular regulators of eryptosis

Death or apoptotic proteins, protein kinases and cell surface receptors are proposed to have a role in the execution or the inhibition of eryptotic pathways. The section below (and Table 2) summarizes available information on such molecular regulators of eryptosis, and highlights the many gaps in knowledge that remain to be filled.

**Table 2:** Molecular regulators of eryptosis.

	**Protein**	**Proposed role in eryptosis**	**References**
Apoptotic proteins	BCL-X_L_/BAK	**Anti-eryptotic** effect observed when BCL-X_L_/BAK complex detected at the plasma membrane and in the absence of serum	Walsh et al., 2002
Kinases	Casein Kinase 1	**Pro-eryptotic**	Kucherenko et al., 2012; Zelenak et al., 2012
	p38 MAP Kinase	**Pro-eryptotic** Phosphorylated upon osmotic shock	Gatidis et al., 2011
	Protein Kinase C	**Pro-eryptotic** Translocates to the membrane upon absence of glucose Direct role on PS exposure	Andrews et al., 2002; de Jong et al., 2002; Klarl et al., 2006
	Janus Kinase 3	**Pro-eryptotic** Phosphorylated/Activated in the absence of glucose	Nosaka et al., 1995; Bhavsar et al., 2011
	AMP-activated Kinase	**Anti-eryptotic** In the absence of glucose	Föller et al., 2009b ; Wang et al., 2010
	cGMP-dependent Kinase I	**Anti-eryptotic**	Föller et al., 2008
	p21-Activated Kinase 2	**Anti-eryptotic** In the absence of glucose	Zelenak et al., 2011
	Raf Kinase	**Anti-eryptotic**	Lupescu et al., 2012
	Mitogen and Stress activated Kinase 1 and 2	**Anti-eryptotic** During hyperosmotic shock and in the absence of glucose	Lang et al., 2015a
Receptor-mediated eryptosis	Glycophorin-C receptor	**Pro-eryptotic** Stimulation leads to PS exposure and hemolysis	Head et al., 2005a
	CD47 receptor	**Pro-eryptotic** Upon conformational change due to oxidative stress or aging Stimulation leads to PS exposure and hemolysis	Head et al., 2005b; Burger et al., 2012
	CD94/Fas receptor	**Pro-eryptotic** Upon oxidative stress (or aging), Fas receptors, Fas ligands, FADD and caspase 8 accumulate within lipid rafts	Mandal et al., 2005

*Molecular modulators of eryptosis published to date. For each factor, their suggested role in eryptosis is indicated. Further investigation is required to confirm involvement in eryptosis and elucidate the exact mechanisms of action*.

### Apoptotic proteins involved in erythrocyte survival

Many cell death proteins are present in mature RBCs, including members of the BCL-2 family such as the pro-apoptotic proteins BAD, BID, BCL10, BAK, BAX, and the anti-apoptotic protein BCL-X_L_ (Walsh et al., 2002; Roux-Dalvai et al., 2008; Lange et al., 2014). These proteins play crucial roles in mitochondria-dependent apoptosis, and are key gatekeepers of viability in nucleated cells.

In healthy nucleated cells, pro-survival BCL-X_L_ and BCL-2 sequester BAX and BAK, while pro-apoptotic BAD is sequestered in a phosphorylation-dependent manner by protein 14-3-3 to repress apoptosis. When apoptotic signals overcome survival signals, the phosphorylation status of BAD changes, causing its release from 14-3-3 and binding to pro-survival BCL-X_L_ and BCL-2, unleashing the apoptotic pore forming BAK and BAX. Perforation of the mitochondrial outer membrane commits the cell to apoptosis: cytochrome c is released, an apoptosome is formed, and effector caspases are activated, ultimately leading to DNA fragmentation, PS exposure and membrane blebbing.

BCL-X_L_ is known to be crucial during haematopoiesis and is particularly strongly expressed during the late stages of erythroblasts maturation (Gregoli and Bondurant, 1997), while expression of BAX and BAK decreases during development of erythroid lineage cells (Gregoli and Bondurant, 1997). In reticulocytes (immature erythrocytes containing a mitochondrion), inhibition of BCL-X_L_ leads to caspase-dependent apoptosis (Delbridge et al., 2017). Similarly, the interaction between BCL-X_L_ and BAK in the plasma membrane is required for the survival of mature RBCs (which lack mitochondria) (Walsh et al., 2002). Indeed, rupture of this interaction using a BAK-mimetic small molecule (resulting in the release of BAK) increases intracellular calcium concentrations and induces PS exposure (Walsh et al., 2002). Interestingly, eryptosis induction through this pathway is inhibited by the addition of serum, suggestive of a key role for serum survival factors in erythrocyte viability. Nevertheless, the role of death proteins within mature erythrocytes, if any, remains to be investigated.

### Protein kinases involved in eryptosis

Phosphorylation is a key regulatory mechanism of all intracellular processes, including apoptosis in nucleated cells. Noteworthy, phosphorylation and other post-translational modifications are possibly even more important in mature erythrocytes, as these cells cannot synthesize *de novo* proteins. Accordingly, numerous protein kinases have been shown to regulate eryptosis. Some, including CK1α, p38 MAP kinase, PKC, and JAK3, stimulate eryptosis; others, such as AMPK, cGKI, PAK2, Raf kinase, and MSK1/2, inhibit this process. The section below summarizes the currently knowledge regarding the mechanisms by which these kinases stimulate or inhibit eryptosis in mature RBCs (also see Table 2).

#### Eryptosis stimulator: casein kinase 1

Casein kinase 1 (CK1) is a serine/threonine protein kinase with seven isoforms in humans, and plays a role in very diverse cellular processes, including cell cycle, gene expression, cytoskeleton modifications, cell adhesion, and modulation of receptor-mediated signaling (Schittek and Sinnberg, 2014). In the context of apoptosis in nucleated cells, CK1α phosphorylates BCL10, BID and Fas-associated death domain (FADD, further described below) (Schittek and Sinnberg, 2014). In RBCs, stimulation of CK1α enhances eryptosis following energy depletion, oxidative stress and osmotic shock (Kucherenko et al., 2012; Zelenak et al., 2012). Conversely, CK1α inhibition blunts eryptosis in these same conditions.

#### Eryptosis stimulator: p38 MAP kinase

p38 mitogen-activated protein kinase (p38 MAPK) is activated by diverse stimuli, such as UV light, heat, osmotic shock and cytokines (Zarubin and Han, 2005). In the context of apoptosis, p38 MAP kinase can have pro- and anti-apoptotic roles depending on the cell type and on the stimulus. In RBCs, p38 MAP kinase is phosphorylated upon osmotic shock, and participates in calcium uptake, PS exposure and cell shrinkage (as p38 MAP kinase inhibition blunts these effects) (Gatidis et al., 2011).

#### Eryptosis stimulator: protein kinase C

Protein Kinase C (PKC) molecules constitute a family of eight serine/threonine protein kinase isoforms that are activated upon increase of Ca^2+^ and diacylglycerol (Mochly-Rosen et al., 2012). Upon activation, PKC is recruited to the membrane where it phosphorylates its substrates. PKC can regulate an important number of processes, including gene expression, protein secretion, cell division and inflammation (Mochly-Rosen et al., 2012). In RBCs, activation of PKC leads to increased calcium intake, cell shrinkage and PS exposure, whereas PKC inhibition prevents calcium entry and PS exposure (Andrews et al., 2002; de Jong et al., 2002). Furthermore, glucose depletion induces PKCα translocation to the membrane, significantly increasing PKC activity and phosphorylation of membrane proteins (Klarl et al., 2006). Interestingly, PS exposure following energy depletion was blunted with PKC-specific inhibitors (Klarl et al., 2006), consistent with a direct role of PKC on PS exposure.

#### Eryptosis stimulator: janus kinase 3

Janus kinase 3 (JAK3) is a cytosolic tyrosine kinase, part of the larger JAK family, which includes JAK1, JAK2, and TYK2. All Janus kinases play a role in signaling triggered by extracellular cytokines and growth factors. Typically, upon binding of a cytokine to its receptors, the receptors multimerize, specific JAK kinases are recruited to the receptor cytosolic domain, and trans-phosphorylate (Rawlings et al., 2004). Janus kinases substrates include STATs (signal transduction and activators of transcription) which then enter the nucleus and regulate transcription. Of relevance, JAK/STAT signaling is crucial for haematopoiesis and JAK3 is predominantly expressed in haematopoietic cells (Nosaka et al., 1995, p. 3). In RBCs, following glucose depletion, JAK3 is phosphorylated on the activating Tyrosine 980 (Nosaka et al., 1995, p. 3). Further, inhibition of JAK3 (or absence of JAK3 in the case of *jak3*^−/−^ mice) blunts the exposure of PS upon glucose depletion, but has no effect on cell size (Bhavsar et al., 2011). Overall, JAK3 is thought to be implicated in membrane scrambling upon energy depletion.

#### Eryptosis inhibitor: AMP-activated kinase

AMP-activated kinase (AMPK) is a serine/threonine kinase that is activated by adenosine monophosphate (AMP) and plays a crucial role in energy homeostasis. Increased concentration of AMP (and a decrease in ATP concentration) is an indication of energy depletion. Upon activation, AMPK switches on catabolic processes that produce ATP, such as glycolysis, and switches off anabolic pathways that consume ATP, such as lipogenesis. Interestingly, mice lacking the AMPK subunit α exhibit erythrocytes with a shorter lifespan compared to their wild-type counterpart (Wang et al., 2010). Additionally, inhibition of AMPK exacerbates eryptosis phenotypes induced by glucose depletion: in an energy depletion context, inhibition of AMPK further increases PS exposure and intracellular calcium, suggesting a role of AMPK in protecting against eryptosis (Föller et al., 2009b).

#### Eryptosis inhibitor: cGMP-dependent kinase I

cGMP-dependent protein kinase I (cGKI) is a serine/threonine kinase activated by cyclic guanosine monophosphate (cGMP), and is implicated in calcium regulation, platelet activation and many other processes (Butt et al., 1994). Noteworthy, mice lacking cGKI display increased levels of eryptosis, as detected by elevated PS exposure, faster RBC clearance and a higher rate of RBC turnover (Föller et al., 2008).

#### Eryptosis inhibitor: p21-activated kinase 2

The p21-activated kinase (PAK) family of serine/threonine kinases comprises 6 members in humans. PAK1, 2 and 3 are effector kinases of the Rho-GTPase CDC42 and Rac, two key cytoskeletal regulatory proteins. PAK2 can also be activated by proteolytic cleavage by caspase 3 during apoptosis (Rane and Minden, 2014). In erythrocytes, PAK2 inhibition leads to PS exposure in the absence of glucose, an effect not exacerbated by the absence of AMPK (*ampk* knock-out mice), suggesting that both these enzymes are acting within the same pathway (Zelenak et al., 2011). Overall AMPK and PAK2 appear to have a pro-survival role during energy depletion.

#### Eryptosis inhibitor: raf kinase

The Rapid accelerated fibrosarcoma (Raf) kinase family of serine/threonine kinases comprises 3 members that act upstream of mitogen-activated protein kinase (MAPK) pathways: A-Raf, B-Raf, and C-Raf. Typically, growth factor receptors activate the Ras GTPase, which activates Raf, which in turn phosphorylates MEK, leading to activation of ERK (Matallanas et al., 2011). Raf is implicated in apoptosis, and a specific inhibitor, Sorafenib, has been developed for cancer treatment (Wilhelm et al., 2004). In erythrocytes, inhibition of Raf with Sorafenib leads to eryptosis, suggesting a protective role of Raf against eryptosis (Lupescu et al., 2012).

#### Eryptosis inhibitor: mitogen and stress activated kinase 1 and 2

MSK1 and MSK2 are serine kinases involved in the MAPK cascade. Once activated by ERK1 and 2, they phospho-activate transcription factors, which then promote cellular proliferation (Wiggin et al., 2002, p. 1). Mice lacking MSK1/2 present a higher turnover of erythrocytes (Lang et al., 2015a). Although in normal conditions, the levels of PS exposure were similar compared to their wild-type counterpart, when MSK1/2-deficient erythrocytes were stressed with hyperosmotic shock or energy depletion, eryptosis (as indicated by PS exposure and cell shrinkage) was significantly enhanced.

Although numerous protein kinases have been shown to be involved in eryptosis, the mechanisms leading to their activation, as well as the specific downstream effectors, are yet to be identified. This represents an important gap in knowledge in the field that requires further investigation.

### Receptor-mediated eryptosis

The third category of molecular regulators of eryptosis encompasses cellular receptors that have the ability to transform extracellular signals into an intracellular cell death cascade. Indeed, similar to apoptosis of nucleated cells, eryptosis can be triggered upon the binding of specific extracellular ligands to various red blood cell surface receptors. RBC receptors that have been shown to trigger eryptosis include glycophorin-C, CD47, and CD95/Fas. We are yet to fully understand the links between activation of these surface receptors and the trigger of eryptosis; and this section summarizes the current knowledge of receptor-mediated eryptosis activation (also see Table 2).

#### Glycophorin-C receptor

Glycophorin-C (GPC) is an important membrane glycoprotein that interacts with the membrane skeleton and plays a role in the maintenance of the shape of the erythrocyte (Tanner, 1993). Interestingly, it has been reported that binding of anti-GPC antibodies leads to PS exposure and hemolysis (Head et al., 2005a).

#### CD47 receptor

Cluster of Differentiation 47 (CD47) is a transmembrane protein that has long been described as a “don't eat-me” signal directed toward the macrophage receptor SIRPα (Oldenborg et al., 2000, p. 47). However, recent studies indicate that CD47 can switch to an “eat-me” signal in aging and oxidative-stressed erythrocytes (Burger et al., 2012, p. 47). Indeed, oxidative stress induces a conformational change of CD47 which, despite retaining binding to the same macrophage SIRPα receptor, will under these conditions induce phagocytosis (Burger et al., 2012). Interestingly, induction of CD47 signaling through binding of its TSP-1 ligand, as well as anti-CD47 antibodies and a specific CD47-binding peptide, trigger PS exposure and hemolysis of RBCs (Head et al., 2005b).

#### CD94/fas receptor

Fas receptor (or CD94 or APO-1) is a well-studied death receptor in the context of apoptosis and other programmed cell death pathways (Schulze-Osthoff et al., 2001). Upon binding of the Fas ligand FasL (either as transmembrane proteins on cytotoxic T lymphocytes, or as soluble proteins) to the Fas receptors on the target cell, the latter form death-inducing signaling complexes (DISC). Aggregation of Fas receptors allows the recruitment of proteins that carry Fas-associated death domain (FADD), leading to the recruitment and activation of caspase 8. Caspase 8 in turn can cleave, and hence activate, BID (with subsequent mitochondria-dependent apoptosis activation) and/or directly activate effector caspase 3. Mandal et al. (2005) showed that Fas receptors, Fas ligands, FADD and caspase 8 accumulate within lipid rafts in old erythrocyte membranes, as well as in oxidative-stressed erythrocytes. Caspase 8 is activated in both old and oxidative-stressed erythrocytes, followed by activation of caspase 3 and reduced activity of flippases (and hence increased PS exposure).

## Studying eryptosis: the tools of the trade

As emphasized above, the molecular events leading to eryptosis remain largely unknown. A number of challenges, inherent to the specific cell type we are considering, explain this gap in knowledge. A key step toward further dissecting the molecular mechanisms of eryptosis relies on robust technical approaches. However, cell death detection techniques relying on nuclear DNA fragmentation or mitochondrial activity cannot be used in this context, for obvious reasons. Nevertheless, a key phenotypic hallmark of eryptosis (and apoptosis) is the cell surface exposure of phosphatidyl serine, which allows flow cytometry to be a key technique commonly used to detect and quantify eryptosis. The binding of fluorescent Annexin V to externalized PS and the cell volume (measured via forward scatter values) are the most common parameters used to assess eryptosis. Additionally, intracellular calcium concentrations can be measured with cell-permeable fluorescent indicators, such as Fluo-3 AM dye. Intracellular ceramide levels can be detected with ceramide-specific antibodies, and the redox state of the cells can be assessed through measurement of GSH/GSSG ratio (reviewed in Pretorius et al., 2016; Jemaà et al., 2017).

Another key hurdle is the study of the RBC proteome given the overwhelming relative amounts of the two most abundant proteins: hemoglobin represents 97% of RBCs' total proteins, and carbonic anhydrase I 1% (Barasa and Slijper, 2014). This implies that all other proteins are present in a much lower relative abundance, and therefore difficult to identify and quantify.

At the genetic level, as RBCs are enucleated, experiments requiring genetic manipulations aimed at investigating the role of specific genes in eryptosis cannot be conducted. However, recent advances in *in vitro* stem cell culture and haematopoietic lineage differentiation, as well as advances in genome editing, offer exciting perspectives to address a number of these issues in the near future (Hockemeyer and Jaenisch, 2016; Caulier et al., 2017). For example, with major advances in *in vitro* stem cell differentiation methods being made, *in vitro* production of large amounts of human mature erythrocytes for transfusion could become a reality (Lapillonne et al., 2010; Caulier et al., 2017). Further, genetic manipulation of stem cells, followed by maturation into erythrocytes, would allow the investigation of molecular events and signaling pathways essential for mature erythrocyte survival. This has indeed been recently explored in the context of indirect areas of RBC research, notably with respect to infection of RBCs with malaria parasites (Egan, 2017; Kanjee et al., 2017).

Finally, other challenges to the study of eryptosis (and erythrocytes in general) include RBC origin, availability, purification, storage conditions and the lack of well-characterized cell lines.

## *Plasmodium*-infected erythrocytes: a fight between life and death

**Figure 3:**
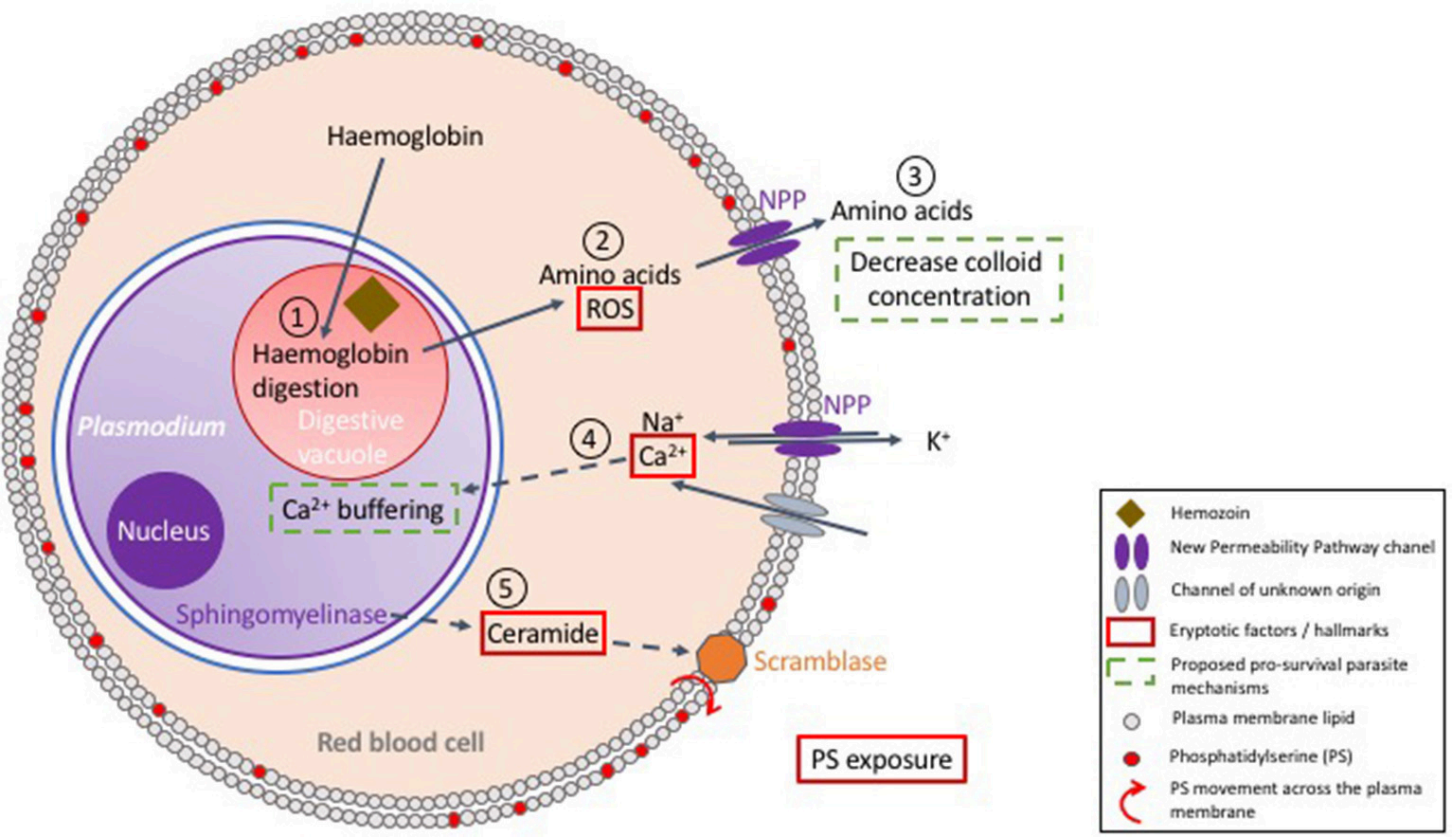
Proposed model of the interactions between *Plasmodium* and its host red blood cell. Upon erythrocyte invasion, *Plasmodium* digests host cell hemoglobin in its digestive vacuole (1). In this process, heme is detoxified by polymerisation into hemozoin and amino acids are utilized for parasite development. The surplus of amino acids is exported to the RBC cytosol (2), and further secreted to the extracellular milieu to decrease colloid concentration (3), which is proposed to enhance survival of the host cell. Digestion of hemoglobin also produces reactive oxygen species (ROS) (2), which enhances eryptosis. New Permeability Pathways (NPPs) and/or other transporters allow Ca^2+^ entry into the red cell cytosol, a key player in triggering eryptosis (4). However, it has been suggested that *Plasmodium* uptakes most of the intracellular calcium in its own cytosol, thus achieving delay of host cell death (4). Nevertheless, active parasite sphingomyelinase has been proposed to break down sphingomyelin, producing ceramide (5), which enhances exposure of phosphatidylserine (PS), and hence recognition and clearance by macrophages.

It is well documented that many pathogens manipulate (i.e., inhibit) the apoptotic pathway of their host cell in order to achieve intracellular survival (viruses: Hay and Kannourakis, 2002; parasites: James and Green, 2004; bacteria: Ashida et al., 2011). In the case of malaria parasites, this has been demonstrated during the infection of liver cells (van de Sand et al., 2005). However, little is known about host-parasite interactions of the erythrocytic stages of infection. Unraveling the molecular regulation of eryptosis is key to our understanding of the interrelationship between *Plasmodium* parasites and their host red cell. *Plasmodium* imposes oxidative stress and induces phosphatidylserine exposure on the host red blood cells, but the clinical outcome arising from PS-exposing *Plasmodium*-infected RBCs is debated. In the context of the tools currently available to study eryptosis, this section reviews published evidence and proposed models that attempt to characterize the interplay between *Plasmodium* and host RBC death mechanisms (also see Figure 3).

### *Plasmodium* imposes oxidative stress on its host cell

During the 48h of its erythrocytic asexual cycle, *Plasmodium falciparum* increases its body mass by up to 32-fold. This requires a considerable supply of nutrients, which the parasite obtains from the digestion of hemoglobin (Zarchin et al., 1986). The ensuing generation of H_2_O_2_ and OH radicals imposes oxidative stress on the host cell (Atamna and Ginsburg, 1993; Ginsburg and Atamna, 1994), which, as discussed above, can lead to erythrocyte's death, which would represent a non-favorable outcome for the parasite (Figure 3).

### Extensive digestion of hemoglobin prevents bursting of the host cell

In addition to the nutrients provided by hemoglobin degradation, parasite survival further relies on the import of nutrients and metabolites from the extracellular milieu, and on a mechanism of waste disposal to the extracellular environment (Kirk, 2001). For this purpose, *Plasmodium* exports parasite encoded transporters to the red cell membrane known as the New Permeability Pathways (NPP) transporters (Kirk, 2001; Huber et al., 2005). Consequently, during trophozoite development, extensive changes take place on the red cell membrane, leading to a dramatic decrease in K^+^, and increase in Na^+^ concentrations in the host cell cytosol to levels resembling those of the extracellular space (Kirk, 2001). The increased cytosolic Na^+^ concentration poses a risk of cell swelling and hemolysis. To prevent this and ensure osmotic stability, the parasite decreases the intracellular colloid osmotic pressure by digesting more hemoglobin than metabolically needed, and exports the excess of amino acids through the NPP transporters, consequently reducing cell swelling and decreasing risks of hemolysis (Lew et al., 2003) (Figure 3).

### *Plasmodium* might sequester intracellular calcium to delay host cell death

In normal conditions, erythrocytes maintain low intracellular Ca^2+^ levels, corresponding to a concentration that is ~ 40,000-fold lower than that of free-Ca^2+^ in the blood plasma (Bogdanova et al., 2013). However, upon *P. falciparum* infection, the erythrocyte's calcium content increases by 10- to 20-fold (Kirk, 2001). Intracellular calcium is crucial for parasite invasion and development (Wasserman et al., 1982) and is thought to be key for many parasite signaling pathways (Enomoto et al., 2012). Interestingly, an increase of free Ca^2+^ in the RBC cytosol is not observed during infection; it is thought that *Plasmodium* accumulates Ca^2+^ in its own cytosol to maintain a low level of free Ca^2+^ in the host cell (Adovelande et al., 1993; Tiffert et al., 2000) (Figure 3). Ca^2+^ sequestration inside the parasite is thought to delay eryptosis, however direct measurements of *in vivo* calcium concentrations in the parasite are required to formally determine whether this is the case. Interestingly, during the liver stage of infection *Plasmodium* sequesters intracellular calcium from hepatocytes, therefore preventing/delaying PS exposure (Sturm et al., 2006). Noteworthy, calcium sequestration mechanisms of erythrocytic stages, if demonstrated, would likely be considered one of the basic foundations for parasite-manipulated eryptosis.

### Increased phosphatidylserine exposure during infection

Despite the fact that *Plasmodium* is proposed to control the levels of free Ca^2+^ in the RBC cytosol, an increase in PS exposure is observed in *Plasmodium*-infected RBCs (iRBCs) when compared to uninfected erythrocytes (uRBCs) (Schwartz et al., 1987). This has been observed not only *in vitro* with *P. falciparum* infection of human RBCs (Schwartz et al., 1987), but also *in vivo* in animal models of malaria infection, such as *P. yoelii* infection of mice (Totino et al., 2010) and *P. knowlesi* infection of monkeys (Joshi et al., 1987). Overall, PS-exposure tends to increase in the mature stages of parasite development (Joshi et al., 1987; Schwartz et al., 1987). A possible explanation for this phenomenon involves the formation of ceramides, a component of sphingomyelin, one of the major structural lipids of cell membranes. Hydrolysis of sphingomyelin, catalyzed by sphingomyelinase, generates ceramide, which, in erythrocytes, leads to PS exposure (see above) even at low cytosolic Ca^2+^ concentrations (Lang et al., 2004) (see Figure 3). Interestingly, erythrocytic forms of *P. falciparum* express a sphingomyelinase that is active during blood stages (Hanada et al., 2000). In line with this observation, a marked decrease of the sphingomyelin content is observed in RBC membranes upon infection by *P. falciparum* (Maguire and Sherman, 1990). Besides the role of ceramide in PS-exposure during infection, it is also interesting that GPC (an erythrocyte receptor able to mediate eryptosis) is used by *P. falciparum* to invade RBCs (Maier et al., 2003), and it has been suggested that this could be the trigger for PS exposure during early stages of parasite development (Head et al., 2005a).

Overall, *Plasmodium* induces eryptosis in the infected cell, by imposing oxidative stress, possibly by producing ceramide, activating GPC-pathways and modulating the erythrocyte intracellular ionic composition. At the same time, *Plasmodium* might also prevent / delay eryptosis and hemolysis by sequestering calcium, and exporting amino acids to the extracellular milieu, as summarized in Figure 3. A balance between induction and prevention (or delay) of eryptosis of the host cell by *Plasmodium* is likely to translate into a successful vs. unsuccessful infection. Although this appears key to our understanding of the clinical outcomes of malaria infections, much is yet to be uncovered in this area.

## *Plasmodium* induces eryptosis of bystander erythrocytes

Further to the eryptosis that *Plasmodium* induces on its own infected cell, it has been observed that malaria infection induces eryptosis of bystander uninfected erythrocytes. In a study using a rodent malaria model, increased numbers of eryptotic uRBCs have been observed in mice 6–7 days post-infection with *P. yoelii* (Totino et al., 2013). Similarly, incubation of human RBCs with serum from patients infected with *P. falciparum* leads to increased PS exposure and decreased cell size of uRBCs (Totino et al., 2014), suggesting that *P. falciparum* is able to induce cell death of non-infected erythrocytes. Interestingly, this has not been observed in the case of *P. vivax* infections, so one cannot exclude that the proinflammatory response common in *P. falciparum* infections, but not in *P. vivax* infections, could be at least partially responsible for this observation (Totino et al., 2014). Further, another *in vitro* study where the role of proinflammatory responses cannot be taken into account, suggests the increase of PS exposure on bystander uRBCs is partially attributed to the presence of methaemoglobin in the extracellular medium. Methaemoglobin is a form of hemoglobin, in which the iron in the heme group is in the Fe^3+^ (ferric) state, not the Fe^2+^ (ferrous) of normal hemoglobin. Upon RBC lysis, hemoglobin is released and oxidized by oxygen to methaemoglobin, which in turn leads to PS exposure of bystander RBCs through oxidative stress (Balaji and Trivedi, 2013). Overall, increased eryptosis of uRBC is thought to be a major contributor of anemia observed during severe malaria infections (Jakeman et al., 1999; Totino et al., 2016), alongside the loss of iRBCs and decreased erythropoiesis (Pathak and Ghosh, 2016). Consequently, a full understanding of the molecular events leading to eryptosis of bystander uRBCs during a malaria infection is crucial to comprehensively address the clinical symptoms of severe anemia in malaria patients.

## A controversial role of eryptosis during malaria infection—is it good or bad?

While it has been established that *Plasmodium* infection contributes to eryptosis of the host cell, the clinical benefit of the phenomenon is debated. On one hand it is argued that PS-mediated clearance of infected cells is favorable to the host. On the other hand it is argued that PS exposure contributes to parasite immune evasion and malaria pathogenesis. This section summarizes the studies that have led to such observations.

### Phosphatidylserine exposure of *plasmodium*-infected erythrocytes implicated in severe disease

It has been argued that PS exposure of iRBCs may play a role in the cytoadherence of *P. falciparum* and enhance tissue sequestration of *P. vivax* (Eda and Sherman, 2002; Totino and Lopes, 2017). Adherence of PS-exposing RBCs to endothelial cells has been demonstrated to occur in various medical conditions and in *in vitro* experiments (Wali et al., 1988; Closse et al., 1999; Bonomini et al., 2002; Setty et al., 2002; Wautier et al., 2011). Therefore, it is plausible that exposure of PS in *Plasmodium*-infected RBCs contributes to adherence and sequestration, as well as to cell aggregation and rosetting (Ho et al., 1991). Consequently, increased PS exposure of iRBCs has two major detrimental effects for malaria patients: sequestered and adherent *Plasmodium*-iRBCs avoid immune clearance; and adherence to the endothelium and other cells induces thrombo-occlusion, which leads to severe disease. Both these events are well characterized in the case of *P. falciparum* infections, and have been to date mainly attributed to the cell surface exposure of PfEMP1, a parasite-derived molecule. Interestingly, in the case of *P*. vivax (which do not express PfEMP1), PS exposure might lead to a more severe course of infection, and there is some evidence that *P. viva*x-iRBCs are able to adhere to endothelial walls and to rosette, due at least in part to phosphatidylserine exposure (Costa et al., 2011; Totino and Lopes, 2017).

### Does phosphatidylserine exposure of *plasmodium*-infected erythrocytes benefit disease outcome?

Despite the data discussed above suggesting that PS exposure in iRBCs is beneficial to parasite survival, two studies propose that eryptosis of *Plasmodium*-infected RBCs is beneficial for the outcome of malaria infection. One study has shown that *P. falciparum* preferentially invades non-eryptotic RBCs (Totino et al., 2010), therefore eryptosis of uninfected RBCs has been interpreted as “a host mechanism to fight malaria” (Totino et al., 2010). The decreased susceptibility of infection of eryptotic cells may in part be attributed to cytoskeleton changes in the host cell (Totino et al., 2010), since actin, spectrin and band 3 proteins, which are crucial for invasion by the parasite (Koch and Baum, 2016), are degraded during eryptosis (Murachi et al., 1981; Schwarz-Ben Meir et al., 1991). In this case, enhanced eryptosis is suggested to correlate with lower parasite burden. Another study proposed that PS-based clearance of ring stage-iRBCs prevents the formation of late-stage parasites, and hence sequestration and associated clinical complications (Föller et al., 2009a). The authors suggest that this may be part of the mechanisms leading to the relative protection against severe malaria in sickle-cell trait, homozygous hemoglobin-C and G6PD-deficiency (Cappadoro et al., 1998; Ayi et al., 2004).

## Inducing eryptosis to treat malaria?

Following from the idea that increased PS exposure of *Plasmodium*-infected RBCs benefits clearance and therefore disease outcome, numerous studies have assessed the effect of eryptosis modulators in the context of malaria. In the section below (and in Tables 2, 3), we summarize the studies that have attempted to date to induce or inhibit eryptosis with the aim of modulating malaria infection, and we further discuss the limitations of such approaches.

**Table 3:** Effect of eryptosis inducers and inhibitors on *P. falciparum in vitro* development.

	**Compound**	**Eryptosis**		**Parasitemia decrease**	**References**
		**uRBC**	**iRBC**	
Eryptosis inducers	Amiodarone 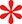 10 μM	ns	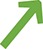	60%	Bobbala et al., 2010b
	Amphotericin B 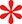 5 μM	ns	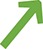	90%	Siraskar et al., 2010
	Aurothiomalate 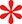 50 μM	ns	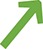	29%	Alesutan et al., 2010
	Azathioprine 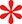 1 μM	ns	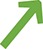	11%	Bobbala et al., 2009
	Anandamide 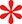 50 μM	ns	ns	70%	Bobbala et al., 2010a
	Chlorpromazine 10 μM	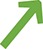	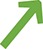	75%	Koka et al., 2008a
	Cyclosporine	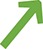 0.01 μM	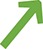 0.001 μM	33% 0.01 μM	Bobbala et al., 2008
	Paclitaxel	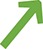 0.1 μM	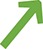 0.001 μM	10% 0.01 μM	Koka et al., 2009
	Lead	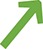 5 μM	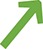 1 μM	ns up to 100 μM	Koka et al., 2007
	L-NAME 10 μM	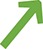	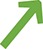	ns up to 100 μM	Koka et al., 2008b
Eryptosis inhibitors	Amitriptyline 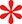 50 μM	ns	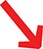	90%	Brand et al., 2008
	Flufenamic acid 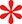 25 μM	ns	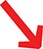 Early stages	19%	Kasinathan et al., 2007

*Eryptosis features of infected red blood cells (iRBC) and bystander uninfected red blood cells (uRBC), as well as parasitemia were measured after 24 or 48 h of treatment with each compound. For clarity purposes, the eryptosis phenotype observed is based only on reported PS exposure measurements. Although not represented in the table, PS exposure of iRBCs was significantly superior to that of uRBCs in all studies. “Parasitemia decrease” indicates the decreased percentage in parasitemia of treated cultures when compared to the untreated control at a given compound concentration (value calculated based on data provided in the original publication). Compounds previously described as inducers or inhibitors or eryptosis (see Table 1) but which do not induce a significant increase or decrease in eryptosis of uRBC in the indicated studies are represented by 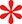. Flufenamic acid induced a decrease of PS exposure of RBC infected with early stage parasites only. ns, not significant; 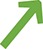, significant increase of PS exposure compared to untreated control; 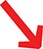, significant decrease of PS exposure compared to untreated control*.

### The effect of eryptosis modulators on the course of a malaria infection

Given the interrelationship between malaria and eryptosis, numerous studies have assessed the effect of eryptosis modulators in the context of malaria (Tables 2, 3 and references therein). Interestingly, various compounds described to induce or inhibit eryptosis in naïve RBCs (Table 1) fail to modulate eryptosis in bystander uRBCs of a *Plasmodium* culture (Table 3, compounds indicated by ^*^). This discrepancy can be attributed to differences between naïve RBCs and *Plasmodium in vitro* culture conditions. Indeed, eryptosis of naïve RBCs is assessed in Ringer solution at 0.4% haematocrit (see references of Table 1), whereas eryptosis of *Plasmodium* cultures is assessed in complete RPMI medium at 4% haematocrit (see references of Table 3). Noteworthy, all but one of the eryptosis inducers tested on malaria parasites (regardless of their ability to induce eryptosis on bystander uRBCs), induce eryptosis of *Plasmodium*-infected RBCs, often at a lower concentration than the one required to induce eryptosis of uRBCs. Further, most of the compounds presented in Table 3 somehow affect parasite viability albeit at variable (and in some cases high) concentrations. Together, these observations provide the main argument in favor of the use of eryptosis inducers as anti-malarial treatments, particularly for those compounds where uRBCs remain unaffected at the concentrations required to affect infected cells. Similar observations were made when using *in vivo* mouse malaria models of infection (Table 4). Five eryptosis inducers have been reported to not modulate eryptosis in bystander uRBCs, while inducing eryptosis of iRBCs. Overall, these *in vivo* studies indicate that inducing eryptosis has a beneficial effect for the host. Indeed, a decrease of parasitemia and increase in mice survival are observed (Table 4). Importantly, few studies measured anemia levels of treated mice, although this is a crucial side effect to consider when testing the use of eryptosis manipulators *in vivo* (Table 4). With respect to the above studies, the possibility has not been excluded that some of the compounds may have off-targets in the parasite. Finally, it is interesting to note that some antimalarial drugs such as artesunate, mefloquine and quinine, have been shown to induce eryptosis (Alzoubi et al., 2014; Bissinger et al., 2015; Mischitelli et al., 2016), raising the question of a possible interference with the eryptotic process to their antimalarial effect.

**Table 4:** Effect of eryptosis inducers on *P. berghei in vivo* development.

***P. berghei in vivo*** **assay**							
	**Compound**	**Eryptosis**		**Parasi-temia decrease**	**Mice survival**	**Anemia effect**	**References**
		**uRBC**	**iRBC**				
Eryptosis inducers	Amiodarone^ 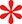 ^	ns	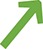	64%	70%	N/A	Bobbala et al., 2010b
	Anandamide^ 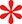 ^	ns	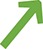	67%	70%	N/A	Bobbala et al., 2010a
	Aurothiomalate^ 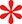 ^	ns	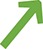	44%	55%	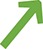	Alesutan et al., 2010
	Dimethylfumarate^ 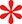 ^	ns	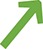	83%	60%	ns	Ghashghaeinia et al., 2010
	Amphotericin B^ 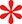 ^	ns	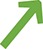	ns	50%	N/A	Siraskar et al., 2010

*Eryptosis compounds were administered to P. berghei-infected mice 8 days post-parasite infection. Eryptosis features of infected red blood cells (iRBC) and bystander uninfected red blood cells (uRBC), as well as parasitemia levels, mice survival outcome, and anemia levels effects were measured every day. For clarity purposes, the eryptosis phenotype observed is based only on reported PS exposure measurements. “Parasitemia decrease” indicates the decreased percentage in parasitemia of treated mice when compared to the untreated control (value calculated based on data provided in the original publication) when the difference reached significance. “Mice survival” indicates the percentage of viable treated mice when untreated controls reached 100% lethality rate. Compounds previously described as eryptotic inducers (see Table 1) that did not induce a significant increase in eryptosis of uRBC in these studies are indicated by ^
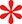
^. ns, not significant; 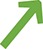, significant increase of PS exposure compared to untreated control; N/A, parameter not discussed in the publication*.

### Inducing eryptosis to treat malaria—is it realistic?

The majority of studies discussed in this review (and summarized in Tables 3, 4) support the notion that enhancing eryptosis of parasitised RBCs represents an attractive anti-malaria strategy. This is based on the hypothesis that exposing RBCs to eryptotic inducers could lead to early clearance of ring stages, preventing trophozoite development and sequestration, and therefore leading to a less severe course of infection. However, it is worth emphasizing that eryptosis inducers that do not specifically target iRBCs, will also increase PS exposure of non-infected RBCs and are therefore likely to lead to anemia. Perhaps the only exception to this, consists in the argument that iRBCs are stressed by the presence of the parasite, therefore the threshold to induce eryptosis may be lower for iRBCs than for uRBCs. Hence the idea of inducing eryptosis selectively (or predominantly) in *Plasmodium*-iRBC may be considered achievable by some authors. It is our view, however, that further work is required to assess the overall potential clinical benefits of eryptosis inducers in the context of a malaria infection. On the other hand, assuming that *Plasmodium* actively inhibits eryptosis of its host cell, an anti-malarial strategy aiming to prevent this inhibition appears more attractive and is discussed in the following section.

## Preventing *plasmodium* from inhibiting eryptosis—a novel approach to treat malaria?

*Plasmodium* interferes with apoptosis of its host cells in the liver of the vertebrate host (Heussler et al., 2006; Kakani et al., 2016) and in the mosquito midgut (Ramphul et al., 2015; Kakani et al., 2016). The parasite thus has a proven track record of manipulating host cell death pathways, and, as alluded to above, also appears to do so during infection of RBCs. Upon RBC invasion, buffering of host cell intracellular calcium and secretion of unused amino acids (Figure 3) have been proposed as the first signs of eryptosis manipulation by the parasite (Adovelande et al., 1993; Tiffert et al., 2000; Kirk, 2001). Consistent with this idea, inhibition of a *P. falciparum* cation-transporting ATPase was shown to induce eryptosis of the iRBC and its rapid clearance *in vivo* (Jiménez-Díaz et al., 2014), as if this inhibition reversed eryptosis silencing. Further, *P. falciparum* activates and relies on human kinases such as mitogen-activated protein kinase kinase (MEK) and p21 activated kinase (PAK), for its development inside RBCs (Sicard et al., 2011). Interestingly, PAK kinases have the ability to inhibit eryptosis in energy depletion conditions, as discussed above (Zelenak et al., 2011) and are often subverted by pathogens for different cellular functions, including manipulation of apoptosis (John von Freyend et al., 2017). Overall, even though there is currently no direct evidence that PAK activation in iRBCs is linked to eryptosis manipulation, it is an exciting hypothesis that opens great perspectives for anti-malaria intervention strategies. Indeed, inhibition of host kinases required by the parasite for survival would ensure disease treatment with smaller risks of emergence of parasite resistance. In addition, host-directed approaches offer the possibility of repurposing human kinase inhibitors already available in the drug-market (Doerig, 2004).

## Conclusions and future directions

Although the complexity of eryptosis regulation in healthy individuals remains to be fully understood, numerous molecular mechanisms have been described to date. Understanding eryptosis regulation is particularly relevant to further our knowledge of *Plasmodium-*erythrocyte interactions. Indeed, manipulation of host cell pathways is a widely used strategy by a multitude of pathogens and offers novel and attractive host-direct therapy opportunities. Manipulation of eryptosis by *Plasmodium* parasites is an emergent area of research that has attracted attention in recent years, and where much is yet to be done. Importantly, host-directed antimalarial therapy offers the considerable advantage of limiting the major pathways toward drug resistance, namely the selection of mutated parasite-encoded targets. However, before this is realized, major gaps in knowledge prevail and need to be addressed. Below, we outline some of the key questions that need to be addressed.

### Potential manipulation of eryptosis by *plasmodium*

Although infection of erythrocytes by *Plasmodium* induces cell death hallmarks, it has been proposed that the parasite inhibits host cell death by buffering intracellular calcium levels (Adovelande et al., 1993), exporting amino acids (Lew et al., 2003), and manipulating host cell kinases (reviewed in Carvalho et al., 2016). Specific molecular mechanisms of calcium sequestration and host ceramide production for example are yet to be elucidated. However, host molecular mechanisms that are shown to be manipulated by the parasite represent exciting opportunities for host-directed therapy. In this context, modulators of eryptosis appear to have a protective effect toward the host in murine models, although possible off-target effects on parasite-encoded factors cannot be excluded, and the influence of these treatments on anemia levels are yet to be determined in most cases.

### Role of ps exposure during *plasmodium* infection

The clinical outcome arising from PS-exposing *Plasmodium*-infected RBCs is controversial. On the one hand, PS-exposure of erythrocytes infected by young parasites is thought to lead to early parasite clearance, hence being favorable to the host (Föller et al., 2009a). On the other hand it is argued that PS exposure enhances the cytoadherence of infected red blood cells, and therefore contributes to parasite immune evasion and malaria pathogenesis (Totino and Lopes, 2017). Although it is clear that *Plasmodium* infection enhances PS exposure of the host erythrocyte, the physiological role of this phenomenon remains to be elucidated and further *in vivo* studies are required to address this question.

### Eryptosis of bystander erythrocytes

Another notable finding in malaria-related eryptosis is the increased eryptosis of uninfected bystander erythrocytes during a malaria infection (Totino et al., 2010, 2013). This is believed to be a key mechanism leading to malaria-induced anemia and the severe outcome of disease (Jakeman et al., 1999). However, the molecular mechanisms underpinning this phenomenon, including a possible role of the immune response, are not understood. There is little doubt that unraveling the mechanism underlying eryptosis of bystander erythrocytes can lead to critical clinical applications in malaria patients suffering from severe anemia.

## Author contributions

CB and TC drafted the manuscript. CD edited and commented on the manuscript.

### Conflict of interest statement

The authors declare that the research was conducted in the absence of any commercial or financial relationships that could be construed as a potential conflict of interest.
