# Erratum: Manipulating Eryptosis of Human Red Blood Cells: A Novel Antimalarial Strategy?

**DOI:** 10.3389/fcimb.2018.00455

**Published:** 2019-01-14

**Authors:** Frontiers Production Office

**Affiliations:** Frontiers Production Office, Frontiers, Lausanne, Switzerland

**Keywords:** malaria, eryptosis, *Plasmodium*, apoptosis, programmed cell death, host-pathogen interaction, host-directed therapy

Due to a production error, two sub-section titles lacked the capitalization of “P” for Plasmodium in “Plasmodium-Infected Erythrocytes: a Fight Between Life and Death" and “Plasmodium Induces Eryptosis of Bystander Erythrocytes.” Secondly, in Table 4 (see below), “uRBC” and “iRBC” column titles should be below “Eryptosis,” as subcolumns. All the “ns” should be below “uRBC,” the green arrows below “iRBC,” and the first column of percentages below “Parasitemia decrease.” Lastly, the latest figures in the final publication were not used.

**Table 4:** Effect of eryptosis inducers on *P. berghei in vivo* development.

***P. berghei in vivo*** **assay**							
	**Compound**	**Eryptosis**		**Parasi-temia decrease**	**Mice survival**	**Anemia effect**	**References**
		**uRBC**	**iRBC**				
Eryptosis inducers	Amiodarone^ 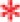 ^	ns	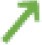	64%	70%	N/A	Bobbala et al., 2010b
	Anandamide^ 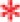 ^	ns	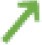	67%	70%	N/A	Bobbala et al., 2010a
	Aurothiomalate^ 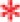 ^	ns	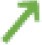	44%	55%	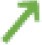	Alesutan et al., 2010
	Dimethylfumarate^ 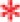 ^	ns	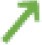	83%	60%	ns	Ghashghaeinia et al., 2010
	Amphotericin B^ 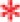 ^	ns	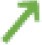	ns	50%	N/A	Siraskar et al., 2010

*Eryptosis compounds were administered to P. berghei-infected mice 8 days post-parasite infection. Eryptosis features of infected red blood cells (iRBC) and bystander uninfected red blood cells (uRBC), as well as parasitemia levels, mice survival outcome, and anemia levels effects were measured every day. For clarity purposes, the eryptosis phenotype observed is based only on reported PS exposure measurements. “Parasitemia decrease” indicates the decreased percentage in parasitemia of treated mice when compared to the untreated control (value calculated based on data provided in the original publication) when the difference reached significance. “Mice survival” indicates the percentage of viable treated mice when untreated controls reached 100% lethality rate. Compounds previously described as eryptotic inducers (see Table 1) that did not induce a significant increase in eryptosis of uRBC in these studies are indicated by ^
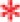
^. ns, not significant; 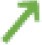, significant increase of PS exposure compared to untreated control; N/A, parameter not discussed in the publication*.

The publisher apologizes for this mistake. The original version of this article has been updated.
